# Case Report: Four Cases of Panayiotopoulos Syndrome Evolving to Juvenile Myoclonic Epilepsy

**DOI:** 10.3389/fneur.2020.591477

**Published:** 2020-12-03

**Authors:** Hideo Enoki, Shinji Itamura, Shimpei Baba, Tohru Okanishi, Ayataka Fujimoto

**Affiliations:** Comprehensive Epilepsy Center, Seirei Hamamatsu General Hospital, Hamamatsu, Japan

**Keywords:** Panayiotopoulos syndrome, juvenile myoclonic epilepsy, self-limited focal epilepsy, idiopathic generalized epilepsy, atypical evolution

## Abstract

Panayiotopoulos syndrome (PS) is a self-limited focal epilepsy appearing in childhood. Seizures in PS are self-limiting and do not usually continue into adulthood. Juvenile myoclonic epilepsy (JME) is the most common type of idiopathic generalized epilepsy, developing around puberty and continuing throughout adulthood. We describe four cases of PS in childhood in which JME developed in adolescence. Age at onset ranged from 4 to 8 years for PS, and 11 to 14 years for JME. JME developed after PS subsided, with the interval between last PS seizure and first JME seizure ranging from 1 to 10 years. No link between the two conditions has previously been described. Since PS is considered to show good prognosis and to be self-limiting, long-term observation has been considered unnecessary. No definitive factors were found to predict future evolution to JME in our series, so longer-term follow-up may be warranted for all PS patients.

## Introduction

Among children, epilepsy syndromes may show changes in the pathological condition within the same patient with development. For example, the age-related evolution of Ohtahara syndrome to West syndrome and Lennox-Gastaut syndrome is well known. In addition to these developmental and epileptic encephalopathies, age-related changes may be observed in other epilepsy syndromes. Whether one causes the other or whether a shared pathology is involved remains unclear. Clarification of the details of age-related evolution could thus contribute to elucidation of the pathophysiology of epilepsies. Here, we report four patients who originally presented with clinical features of Panayiotopoulos syndrome (PS) in childhood and later showed development of juvenile myoclonic epilepsy (JME) in adolescence.

PS is one of the most common self-limited focal epilepsies appearing only in children, with a peak age at onset of 4–5 years old (1, 2). The clinical manifestations of PS are unique. The core symptoms comprise autonomic manifestations, eye deviation, and impairment of consciousness (1). Among these, the most characteristic is the presence of autonomic symptoms, mainly in the form of ictal emesis, and often accompanied by pallor. Ictal non-autonomic symptoms may include clonic convulsions of the face and extremities, often on one side. Seizures typically start during sleep (1, 3). Seizure duration is usually long (4), with almost half lasting >30 min (1–3). Status epilepticus (SE) is usually non-convulsive, with main symptoms in the form of autonomic manifestations. Inter-ictal electroencephalography (EEG) of PS demonstrates significant variability. Occipital spikes represent the main EEG abnormality, but spike discharges may appear anywhere (2).

The prognosis of PS is quite favorable. A quarter of patients with PS experience only a single seizure, and half have two to five seizures (2). Although PS often enters remission within 1–2 years of onset (2–4), evolution to other forms of epilepsy may occur during childhood. A typical example of this evolution is self-limited epilepsy with centrotemporal spikes, formerly called “benign epilepsy with centrotemporal spikes” (BECT). An estimated 13% of patients with PS develop BECT later in childhood (2). BECT is an age-related epilepsy, with remission before 16 years old. Patients with PS, irrespective of whether they develop BECT, thus typically achieve release from epilepsy before reaching adulthood. Textbooks state that the risk of subsequent epilepsy in adult life among patients with a history of PS appears to be no higher than that in the general population (2–4).

JME is the most common type of idiopathic generalized epilepsy, characterized by myoclonic seizures (predominantly in the arms) appearing around puberty. Most patients exhibit generalized tonic-clonic seizures (GTC), and some also experience absence seizures.

Evolution from PS to JME in the same patient does not appear to have been described previously. Similarly, PS has not been reported in the past history of patients with JME. As a result, no link between the two conditions is known. Both syndromes are classed as idiopathic epilepsies, but are classified into totally different syndromes, with PS as a focal epilepsy and JME as a generalized epilepsy. This evolution thus means that the pathological type can change from focal to generalized epileptogenesis with age. In this respect, we have a strong interest in this phenomenon. The present report is the first to describe this rare condition.

## Patient Selection

We conducted a retrospective study of patients with epilepsy who visited Seirei Hamamatsu General Hospital for the first time between January 2004 and December 2014. We collected 1,189, pediatric patients ≤ 15 years old, and identified 56 patients (4.7%) with PS.

The diagnostic criteria for PS we adopted were as follows (2, 4, 5):

- Age at onset between 1 and 14 years;
- Autonomic manifestations, and/or simple motor focal seizures followed or not by impairment of consciousness with or without secondary generalization;
- Functional occipital and extraoccipital spikes alone or combined on interictal EEG, or a normal EEG;
- Normal neurological and mental statuses; and
- Normal results from brain imaging.

The 56 patients we identified were monitored up to July 2020. Ten patients were transferred to another hospital within 1 year due to relocation, etc. Two patients dropped out and were unable to be followed. We then performed long-term observations of 44 patients for 4–13 years (mean, 9.2 years). Of these, 11 patients evolved from PS to other epileptic conditions, comprising 4 cases of JME, 4 cases of epileptic encephalopathy with continuous spikes and waves during sleep (CSWS), 1 case of BECT, 1 case of temporal lobe epilepsy, and 1 case of single unprovoked seizure.

The diagnosis of JME was based on the International League Against Epilepsy (ILAE) classification (6) and recent reviews (7, 8). We encountered four patients who met the diagnostic criteria for PS in childhood and developed JME in adolescence.

## Case Presentation

We conducted retrospective long-term observations for the four patients and analyzed clinical features as summarized in Table 1.

**Table 1:** Clinical information of the 4 cases.

		**Case 1**	**Case 2**	**Case 3**	**Case 4**
Sex		F	F	M	F
Family history, convulsive disorders		Fs, mother	None	Fs, mother	TLE, sister
Peri- or postnatal disorders		Cesarean section due to pelvic position	None	None	Low birth weight
Past history, convulsive disorders		Febrile SE	None	Fs	Fs
PS	Age at onset	5 y 6 m	4 y 2 m	8 y 6 m	8 y 10 m
	Circadian distribution of sz	Sleep	Sleep	Awake	Awake
	Total no. of sz	9	1	3	6
	No. of prolonged sz (5–30 min)	4	1	1	4
	No. of SE episodes (> 30 min)	4	0	0	1
	EEG discharges	Occipital	Occipital	Occipital	Generalized, frontopolar, OIRDA, FIRDA
	Head MRI	Normal	Normal	Normal	Normal
	AED	VPA, CLB	VPA	VPA	CBZ, LTG
	Age at last sz	7 y 4 m	4 y 2 m	8 y 10 m	10 y 6 m
	Age when AED stopped	13 y 6 m	12 y 6 m	11 y 5 m	12 y 5 m
JME	Age at onset of myoclonic sz	14 y 6 m	14 y 5 m	11 y 8 m	12 y 2 m
	Age at onset of GTC	14 y 6 m	14 y 7 m	12 y 1 m	12 y 7 m
	EEG discharges	Wave-and-spike phantom	Generalized	Generalized	Generalized, PPR
	Head MRI	Normal	Normal	Normal	Normal
	AED	LEV	VPA	VPA	LTG
	Age at last GTC	14 y 6 m	14 y 7 m	12 y 1 m	20 y 3 m
Interval between last PS and first JME sz		7 y 2 m	10 y 3 m	2 y 3 m	1 y 8 m
Reported neurobehavioral disorders^*^		None	Borderline intellectual function (IQ 72)	None (IQ 110)	None
Present age		17 y 7 m	16 y 7 m	16 y 7 m	20 y 5 m

*sz, seizure/s; SE, status epilepticus; AED, antiepileptic drug; GTC, generalized tonic-clonic seizure; PS, Panayiotopoulos syndrome; JME, juvenile myoclonic epilepsy; Fs, febrile seizure; TLE, temporal lobe epilepsy; OIRDA, occipital intermittent rhythmic delta activity; FIRDA, frontal intermittent rhythmic delta activity; PPR, photoparoxysmal response; VPA, valproic acid; CLB, clobazam; LEV, levetiracetam; CBZ, carbamazepine; LTG, lamotrigine*.

* *Evaluated in adolescence*.

### Case 1

The patient was a girl who had suffered from six episodes of febrile seizure since 1 year old, one of which lasted for 1 h. We performed EEG four times from 2 to 4 years old, and none revealed epileptic discharges. Developmental history was normal. Her mother had a history of febrile seizure, but all other family members were reportedly free from seizure disorders.

At 5 years and 6 months old, she was transported by ambulance and admitted to our hospital due to impairment of consciousness. Her parents reported sudden vomiting that started during a nap while traveling in a car, and repeating several times. She woke from the nap, and was initially able to speak, but gradually became unconscious. Her eyes then deviated to the right, her complexion became pale, and her limbs became flaccid. When the patient arrived at our hospital 70 min after seizure onset, she was still lethargic, but her eyes were closed without deviation. The emergency room doctor decided that the seizure had already stopped and she was in a state of post-ictal drowsiness. EEG revealed epileptic spikes in the left occipital region, whereas magnetic resonance imaging (MRI) showed no abnormalities. PS was then diagnosed. After observing the course without treatment, the patient experienced another seizure 9 months later. This time, the seizure evolved to left hemiconvulsions with eyes deviating to the left. Medication started with valproic acid (VPA), with later addition of clobazam, but neither achieved resolution of seizures. In total, she experienced nine seizure events equivalent to PS by 7 years and 4 months old, most of which were lengthy seizures lasting >5 min or SE >30 min. The longest seizure lasted 60 min before stopping on intravenous injection of diazepam. Although she experienced no further PS seizures after 7 years old, EEG spike discharges were consistently observed. Medication was continued in accordance with the wishes of the parents until 13 years 6 months old, by which time the EEG abnormalities had disappeared.

At 14 years 6 months old, the patient noticed twitching in the arms, particularly on waking. That same month, she suffered GTC right after waking. EEG showed a 6-Hz wave-and-spike phantom, and results of MRI were normal. Based on the seizure symptomology and laboratory findings, we diagnosed JME. Levetiracetam (LEV) was started, and completely eliminated seizures without adverse effects. The patient is currently 17 years 7 months old and has shown no further symptoms.

### Case 2

**Figure 1:**
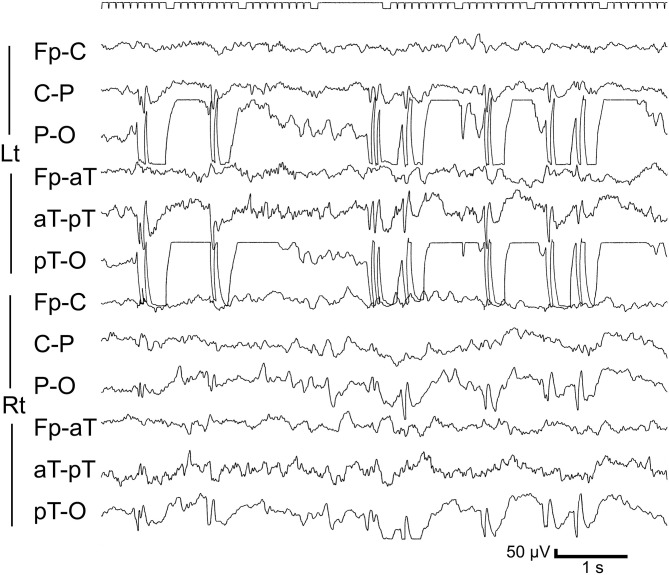
Representative EEG from Case 2 during the PS period. Sleep record at 5 years 9 months old. Upon medication with valproic acid. Frequent spike-and-waves are seen in the left occipital region.

A 4-year-2-month-old girl was transported by ambulance to our hospital due to a first episode of afebrile convulsions. During a nap, she started to vomit and turned pale, and proved unresponsive to her parents' calls. Right upper limb convulsions then began. The whole course of the seizure was about 8 min. No information was available on whether her eyes showed a deviation during the seizure. EEG demonstrated a left occipital spike-and-wave pattern. Results of MRI were normal. Although we advised observation of her condition without medication, VPA was started based on the strong desire of the parents. No subsequent seizures appeared, but left occipital spike-and-wave discharges persisted for a long time (Figure 1). VPA was discontinued at 12 years 6 months old, after confirming the disappearance of EEG abnormalities.

Two years later, she suffered myoclonic jerks and a GTC. EEG showed generalized polyspikes and waves. MRI was again performed, but demonstrated no abnormalities. We diagnosed JME and explained the need for medication throughout adulthood. Considering side effects of VPA on women of childbearing potential, we recommended a new-generation antiepileptic, such as LEV or lamotrigine (LTG). However, the patient and her parents opted to use the same drug she had used before. VPA was restarted at a low dose, and achieved total seizure control.

### Case 3

An 8-year-old boy suffered from three episodes of afebrile seizure that occurred while awake. Symptoms consisted of repeated vomiting and impairment of consciousness. One of the episodes was accompanied by left upper limb convulsions and deviation of the eyes to the left. Seizure duration ranged from 3 to 10 min. Right occipital spike discharges were confirmed on EEG. Results of MRI were normal. VPA was started after the third seizure, and achieved control of seizures. The drug was discontinued at 11 years old, after confirming complete normalization of EEG findings.

However, after stopping the drug, generalized spike-and-wave discharges appeared on EEG. After a while, he started experiencing myoclonic jerks. In addition, at 12 years 1 month old, he suffered a GTC. VPA was restarted, and effectively stopped the seizures.

### Case 4

**Figure 2:**
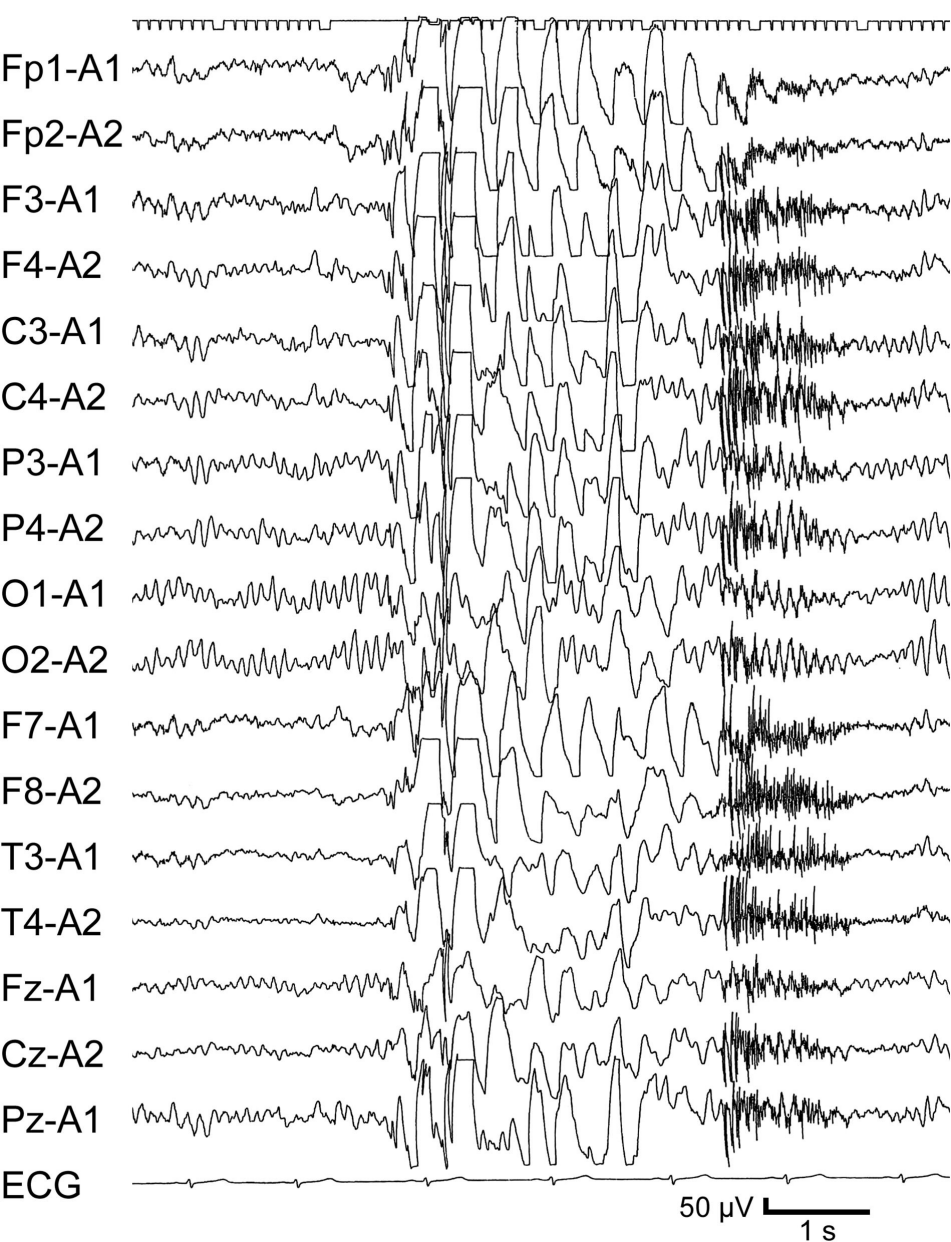
Interictal EEG from Case 4 when JME developed. Awake record at 12 years 7 months old, on no medication. Generalized polyspikes-and-waves are apparent.

A 12-year-7-month-old girl was referred to our outpatient clinic due to an episode of GTC. She had a history of focal epilepsy that started at 8 years old. Focal seizures were characterized by initial vomiting, followed by deviation of the eyes and impairment of consciousness without convulsions of the extremities. She had been administered carbamazepine (CBZ) and LTG. At 12 years 2 months old, she noticed twitching in the arms. She discontinued the medication without medical advice at 12 years 5 months old. GTC subsequently occurred at 12 years 7 months old. EEG revealed frequent generalized polyspike-and-wave discharges (Figure 2) and photoparoxysmal responses. LTG effectively improved the EEG abnormalities, but GTCs did not completely cease and appeared triggered by menstruation or a lack of sleep.

## Discussion

We have presented here a series of four patients who developed PS in childhood with evolution to JME in adolescent. Although all patients fulfilled the diagnostic criteria for PS, age at onset in Cases 3 and 4 was relatively high (8 years old) and seizures in these patients appeared while awake. As mentioned earlier, peak age at onset is 4–5 years and seizures tend to occur during sleep in PS. Differential diagnosis would thus be important in these cases. Idiopathic etiology, focal seizures while awake, and onset age at 8 years lead to consideration of childhood occipital epilepsy of Gastaut. In that syndrome, the main seizure symptom is visual hallucinations, with frequent complaints of post-ictal headache. No such symptoms were reported in Cases 3 and 4, and EEG in the latter case showed no occipital spikes. A diagnosis of Gastaut-type epilepsy thus could not be given.

Transition of PS to JME has not been reported previously and is clearly rare. The characteristics of such patients thus require investigation. We therefore compared our series with previous PS case studies of large groups. We identified two studies (5, 9), in which the evolution of PS to JME did not appear, and summarized the cases in Table 2. We did not perform statistical analyses, because the number of cases in our series was small. However, the frequency of a history of febrile seizures seemed high in our series. No characteristic differences were seen in other factors, such as sex, family history, age at onset, circadian distribution, seizure semiology, SE, single seizure, and EEG findings. Of course, a history of febrile seizure alone cannot determine the prognosis. We were thus unable to identify any factors predicting future evolution.

**Table 2:** Comparison with clinical studies of Panayiotopoulos syndrome.

	**Caraballo et al. (5)**	**Specchio et al. (9)**	**Present series**
Number of patients	192	93	4
Sex, %	Girls 44, boys 56	Girls 55.9, boys 44.1	Girls 75, boys 25
Family history, %	Epilepsy 31.3, Fs 13.5	Epilepsy/Fs 45.2	Epilepsy 25, Fs 50
Past history of Fs, %	28.5	6.5	75
Age at onset, y (median)	1.7–12.5 (4.5)	1.1–8.6 (4.1)	4–8 (6.5)
Circadian distribution of sz, %	Sleep 97.4 Awake 30.7	Sleep 69.9 Awake 17.2 Awakening 12.9	Sleep 50 Awake 50
Sz semiology, %			
Ictal emesis	82.3	77.4	100
Eye deviation	88.5	73.1	75^*^
Impairment of consciousness	78.1	89.2	100
SE, %	36.4	54.8	50
Single sz, %	44.2	33.1	25
Occipital EEG discharges, %	75	76.1	75

*Fs, febrile seizure; sz, seizure/s; SE, status epilepticus*.

* *Details unknown in remaining 25%*.

Aside from JME, evolution from PS to other epilepsy syndromes has been reported in the literature. The representative condition for this situation is BECT (5, 10). This pathology develops after a seizure-free period, or coexists with PS. Other than BECT, the visual seizures of occipital epilepsy might arise in rare cases (3, 4). All these evolutions result in the development of focal seizures.

Aside from these focal seizures, generalized seizures have been described in PS as an atypical evolution in very rare cases, such as absence seizures (5, 11–14), negative myoclonus (5), and myoclonic seizures (13). With these atypical evolutions, worsening of EEG as characterized by diffuse spike-and-wave discharges was shown. As a consequence of the worsening of EEG findings, encephalopathy related to status epilepticus during slow sleep (ESES) or epileptic encephalopathy with continuous spikes and waves during sleep (CSWS) has been reported during the course of PS (5, 15). However, none of our cases showed an EEG pattern consistent with CSWS. Our series appears totally distinguished from the atypical evolutions of PS reported in the literature because our cases demonstrated markedly different findings from EEG.

These atypical evolutions of PS are extremely rare and exceptional. As for the issue of evolution from PS to generalized epilepsy, Caraballo et al. found that 1 of 160 patients with PS developed typical absence seizures and concluded that the absence seizures might have merely been coincidental (11). However, they later found a past history of PS in 3 of 203 patients with childhood absence epilepsy (CAE) (14). This raised the question of whether the relationship between PS and CAE is coincidental, or is a common pathophysiology involved in focal and generalized epilepsy. They hypothesized a close genetic relationship between both epileptic syndromes, while leaving the possibility of mere coexistence. Both CAE and JME are generalized epilepsies. The issue raised by Caraballo et al. in CAE is closely related to the concept of evolution from PS to JME. We speculate that the evolution described here is unlikely to represent coincidence, as the present series collected four cases, not just one. Clarification of this hypothesis will require the accumulation of a larger number of cases in future genetic studies.

From another perspective, the influences of pharmacotherapies should be considered for the atypical evolutions. Kikumoto et al. reported a patient with PS who developed absence and myoclonic seizures due to exacerbation by CBZ (13). However, our cases were unlikely to have involved drug-induced exacerbation. In Cases 1–3, medications had already been discontinued before onset of JME. In the remaining case, myoclonic seizures appeared during treatment with CBZ and LTG, but discontinuation of these agents did not achieve seizure cessation, and GTC appeared during the drug-free period. Neither CBZ nor LTG was thus considered a trigger for JME development in Case 4.

The evolutions of PS previously described in the literature, namely BECT, occipital epilepsy, ESES or CSWS, CAE, and exacerbations due to drugs are all transient pathologies. All are age- or drug-dependent and can remit eventually. However, JME persists throughout adulthood. In that respect, the evolution of PS to JME appears distinct from any previously reported evolutions of PS.

Our series suggests the evolution from self-limited focal epilepsy to JME represents a natural course, rather than an anthropogenic effect such as drug-induced exacerbation. The commonalities between focal epilepsy and JME have also been reported in other epilepsy syndromes, such as idiopathic photosensitive occipital epilepsy (IPOE). IPOE is a focal epilepsy that develops from late childhood to adolescence and has been reported to overlap with JME (16). Both have been suggested to involve a common genetic determinant (16). Our series provides a contribution to the literature on the spectrum of childhood-onset focal epilepsies that may evolve to JME.

In this study, long-term observation revealed that 11 of the 56 PS cases evolved to other epileptic pathologies, including JME. This rate of evolution was surprisingly high. Given that epileptic conditions in these cases were not actually “self-limited” and that the rate of evolution to other conditions was so high, the prognosis of so-called “self-limited epilepsies” needs to be reassessed. Further long-term, observational studies of large groups are thus required.

## Concluding Remarks

Although the background pathology in the evolution cannot yet be clarified from this case series alone, the purpose of this paper was to show the existence of such rare cases and to recommend that clinicians pay attention after PS. Since PS is known to display a good prognosis, the need for long-term observation has not been recognized. In our series, JME appeared even 10 years after the end of PS. We now understand that PS can evolve to a specific epileptic state and definitive factors predicting this future evolution have yet to be identified. Further accumulation of data with long-term follow-up is needed for all PS patients to clarify the pathophysiology of this evolution.

## Data Availability Statement

The original contributions presented in the study are included in the article/supplementary materials, further inquiries can be directed to the corresponding author/s.

## Ethics Statement

The studies involving human participants were reviewed and approved by Ethics board at Seirei Hamamatsu General Hospital. Written informed consent to participate in this study was provided by the participants' legal guardian/next of kin.

## Author Contributions

HE and AF contributed to the conceptualization, drafting, and revision of the study. SI, SB, and TO contributed to the acquisition and interpretation of clinical information and EEG data. All authors contributed to the article and approved the submitted version.

## Conflict of Interest

The authors declare that the research was conducted in the absence of any commercial or financial relationships that could be construed as a potential conflict of interest.

## References

[1] Fejerman N Early-onset benign childhood occipital epilepsy (Panayiotopoulos type). In: Engel J Pedley T editors. Epilepsy: A Comprehensive Textbook. 2nd ed Philadelphia, PA: Lippincott Williams & Wilkins (2008). p. 2379–86.

[2] Panayiotopoulos CP Panayiotopoulos syndrome. In: Panayiotopoulos CP editor. A Clinical Guide to Epileptic Syndromes and Their Treatment. 2nd ed. Based on the ILAE Classifications and Practice Parameter Guidelines. London: Springer-Verlag (2010). p. 347–56.

[3] Demirbilek V Bureau M Çokar Ö Panayiotopoulos CP Self-limited focal epilepsies in childhood. In: Bureau MGP Dravet C Delgado-Escueta A Guerrini R Tassinari CA Thomas P editors. Epileptic Syndromes in Infancy, Childhood and Adlescence. 6th ed. Montrouge: John Libbey EUROTEXT (2019). p. 219–60.

[4] Ferrie C Caraballo R Covanis A Demirbilek V Dervent A Kivity S Panayiotopoulos syndrome: a consensus view. Dev Med Child Neurol. (2006) 48:236–40. 10.1017/S0012162206000508 16483404

[5] Caraballo R Cersósimo R Fejerman N. Panayiotopoulos syndrome: a prospective study of 192 patients. Epilepsia. (2007) 48:1054–61. 10.1111/j.1528-1167.2007.01085.x 17442007

[6] Scheffer IE Berkovic S Capovilla G Connolly MB French J Guilhoto L ILAE classification of the epilepsies: position paper of the ILAE Commission for Classification and Terminology. Epilepsia. (2017) 58:512–21. 10.1111/epi.13709 28276062 PMC5386840

[7] Baykan B Wolf P. Juvenile myoclonic epilepsy as a spectrum disorder: a focused review. Seizure. (2017) 49:36–41. 10.1016/j.seizure.2017.05.011 28544889

[8] Wolf P Yacubian EM Avanzini G Sander T Schmitz B Wandschneider B. Juvenile myoclonic epilepsy: a system disorder of the brain. Epilepsy Res. (2015) 114:2–12. 10.1016/j.eplepsyres.2015.04.008 26088880

[9] Specchio N Trivisano M Di Ciommo V Cappelletti S Masciarelli G Volkov J. Panayiotopoulos syndrome: a clinical, EEG, and neuropsychological study of 93 consecutive patients. Epilepsia. (2010) 51:2098–107. 10.1111/j.1528-1167.2010.02639.x 20528983

[10] Caraballo R Cersósimo R Fejerman N Idiopathic partial epilepsies with rolandic and occipital spikes appearing in the same children. J Epilepsy. (1998) 11:261–4 10.1016/S0896-6974(98)00014-0

[11] Caraballo RH Sologuestua A Grañana N Adi JN Cersósimo RO Mazza E. Idiopathic occipital and absence epilepsies appearing in the same children. Pediatr Neurol. (2004) 30:24–8. 10.1016/S0887-8994(03)00409-0 14738945

[12] Ferrie CD Koutroumanidis M Rowlinson S Sanders S Panayiotopoulos CP. Atypical evolution of Panayiotopoulos syndrome: a case report. Epileptic Disord. (2002) 4:35–42. Available online at: https://www.jle.com/fr/revues/epd/e-docs/atypical_evolution_of_panayiotopoulos_syndrome_a_case_report_published_with_video_sequences_._110061/article.phtml 11967178

[13] Kikumoto K Yoshinaga H Oka M Ito M Endoh F Akiyama T. EEG and seizure exacerbation induced by carbamazepine in Panayiotopoulos syndrome. Epileptic Disord. (2006) 8:53–6. Available online at: https://www.jle.com/download/epd-268134-3365-eeg_and_seizure_exacerbation_induced_by_carbamazepine_in_panayiotopoulos_syndrome-a.pdf 16567326

[14] Caraballo RH Fontana E Darra F Bongiorni L Fiorini E Cersosimo R Childhood absence epilepsy and electroencephalographic focal abnormalities with or without clinical manifestations. Seizure. (2008) 17:617–24. 10.1016/j.seizure.2008.03.009 18524634

[15] Oguni H Hirano Y Nagata S. Encephalopathy related to status epilepticus during slow sleep (ESES) as atypical evolution of Panayiotopoulos syndrome: an EEG and neuropsychological study. Epileptic Disord. (2020) 22:67–72. 10.1684/epd.2020.1128 32020894

[16] Taylor I Marini C Johnson MR Turner S Berkovic SF Scheffer IE. Juvenile myoclonic epilepsy and idiopathic photosensitive occipital lobe epilepsy: is there overlap? Brain. (2004) 127:1878–86. 10.1093/brain/awh211 15201194

